# Early Neonatal Hyperglycemia, Risk Factors, and Adverse Outcomes in Extremely Preterm Infants: A Propensity-Matched Cohort Study

**DOI:** 10.3390/children13030387

**Published:** 2026-03-10

**Authors:** Safaa M. G. A. Alsayigh, Nuha Nimeri, Alaa Almashhadani, Amna Abdelgadir Mohamed, Omar Haidar, Muhammed Talha Hepsen, Ashraf Gad

**Affiliations:** 1 Neonatal Intensive Care Unit, Women’s Wellness and Research Center, Hamad Medical Corporation, Doha 3050, Qatar; nnimeri@hamad.qa (N.N.); v-aalmashhadani@hamad.qa (A.A.); agad2@hamad.qa (A.G.); 2 Pediatric Department, Weill Cornell Medicine, Doha P.O. Box 24144, Qatar; 3 Department of Pediatrics, Hamad General Hospital, Hamad Medical Corporation, Doha 3050, Qatar; v-aahmed13@hamad.qa (A.A.M.); ohaidar02@gmail.com (O.H.); talhahepsen@gmail.com (M.T.H.)

**Keywords:** extremely preterm infants, neonatal hyperglycemia, ROP, ventilator-associated pneumonia, propensity score matching

## Abstract

**Highlights:**

**What are the main findings?**

- Early neonatal hyperglycemia in extremely preterm (EP) infants is linked to higher rates of ventilator-associated pneumonia, prolonged mechanical ventilation, severe retinopathy of prematurity, and a trend toward increased moderate-to-severe bronchopulmonary dysplasia, even after propensity score matching.
- These associations suggest that early neonatal hyperglycemia may independently contribute to adverse short-term outcomes in this high-risk population.

**What are the implications of the main findings?**

- Early recognition of neonatal hyperglycemia may help identify EP infants at higher risk for respiratory and ophthalmological morbidities, suggesting a potential target for closer monitoring and future interventional studies.

**Abstract:**

Background: Neonatal hyperglycemia is a common metabolic complication in extremely preterm (EP) infants; however, early risk factors and associated outcomes remain incompletely defined. Objective: To evaluate the association between neonatal hyperglycemia in the first postnatal week and key neonatal morbidities including early neurodevelopmental risk in EP infants. Methods: We conducted a retrospective cohort study of EP infants born in 2018–2019 at the Women’s Wellness and Research Center. Neonatal hyperglycemia was defined as a blood glucose level > 8.3 mmol/L. Maternal factors, delivery room interventions, early physiological markers, neonatal morbidities, and follow-up outcomes were compared. Propensity score matching was applied to balance the baseline demographic and perinatal differences. Results: Among 225 EP infants, 131 (58.2%) developed neonatal hyperglycemia in the first week (mild, 21.4%; moderate, 42%; severe, 36.6%). Before matching, infants with neonatal hyperglycemia had lower gestational age and birth weight and required more delivery-room surfactant, and their mothers had lower rates of premature rupture of membranes. After matching, neonatal hyperglycemia was associated with higher rates of ventilator-associated pneumonia (1.45 vs. 0.37; IRR 6.2, 95% CI 1.4–27.6), longer duration of invasive ventilation (19.8 ± 25.3 vs. 8.9 ± 24.8 days; mean difference −10.9 days; *p* = 0.042), higher postnatal steroid exposure (18.2% vs. 5.5%; OR 4.6, 95% CI 1.6–14.4; *p* = 0.040), and severe retinopathy of prematurity (ROP) (21.6% vs. 6.4%; OR 4.0, 95% CI 1.0–15.5; *p* = 0.032). A trend toward moderate-to-severe bronchopulmonary dysplasia was observed (33.3% vs. 15.9%; *p* = 0.054). Mortality did not differ significantly between groups; however, among non-survivors, age at death was higher in the neonatal hyperglycemia group. Conclusions: In EP infants, early neonatal hyperglycemia is associated with higher respiratory morbidity and severe ROP even after propensity score matching. These findings support neonatal hyperglycemia as a clinically relevant early risk marker and justify further prospective and interventional studies.

## 1. Introduction

Neonatal hyperglycemia is one of the most common metabolic disorders in extremely preterm (EP) infants [1]. This results from multiple factors, including insulin resistance, stress-related catecholamine release, immature pancreatic beta-cell function, and exogenous glucose administration [2]. Incidence rates vary widely, from 20% to 80%, depending on the gestational age, illness severity, and nutritional management [3,4]. Neonatal hyperglycemia has been associated with adverse outcomes such as necrotizing enterocolitis (NEC), bronchopulmonary dysplasia (BPD), retinopathy of prematurity (ROP), sepsis, and increased mortality [5,6,7,8]. However, these associations are confounded by the biological vulnerability of this population, making it unclear whether neonatal hyperglycemia is an independent risk factor or merely a marker of disease severity [1,9,10,11].

Recent studies have confirmed that hyperglycemia in EP infants is linked to significant short- and long-term morbidities. It is associated with increased mortality, intraventricular hemorrhage (IVH), and ROP, particularly when blood glucose levels exceed 10 mmol/L or when hyperglycemia is prolonged. Observational data also suggest associations with chronic lung disease and neurodevelopmental impairment, although causality remains uncertain. Pathophysiologically, neonatal hyperglycemia reflects a combination of insulin resistance and impaired insulin secretion, both of which are strongly influenced by prematurity. Hyperglycemic neonates exhibit reduced endogenous insulin production, and exogenous insulin therapy does not suppress pancreatic secretion, underscoring the complexity of metabolic immaturity [1,11,12]

Neonatal hyperglycemia may also contribute to oxidative stress, inflammation, and endothelial dysfunction, mechanisms implicated in ROP, BPD, and increased infection risk [1,13]. Furthermore, prolonged hyperglycemia may disrupt retinal vascular development, increasing the likelihood of ROP and the need for intervention. Long-term follow-up studies show that each day of blood sugar level > 8 mmol/L is associated with lower intelligence scores and worse motor outcomes at 6.5 years of age, while insulin treatment does not appear to improve neurodevelopmental outcomes [14]. Despite these associations, optimal thresholds for intervention and management strategies remain controversial, as lowering glucose infusion risks calorie deficits and growth impairment, while insulin therapy increases hypoglycemia risk [14,15,16,17].

While associations exist, it remains unclear whether neonatal hyperglycemia is an independent risk factor or a proxy for overall immaturity and illness severity in this population. To isolate the effect of early neonatal hyperglycemia, we employed propensity score matching to control key perinatal confounders and assess its independent association with morbidity in EP infants.

## 2. Materials and Methods

### 2.1. Study Design, Setting, and Population

We conducted a retrospective propensity-matched cohort study in the Level III Neonatal Intensive Care Unit (NICU) of the Women’s Wellness and Research Center, Hamad Medical Corporation, Doha, Qatar. This tertiary referral center manages approximately 17,000 deliveries annually. Approximately 12–15% of live births require NICU admission.

All live-born infants with a gestational age (GA) < 28 weeks who were admitted to the NICU within 24 h of birth between January 2018 and December 2019 were eligible. Outborn infants who were admitted more than 24 h after birth, infants who died in the first 12 h of age, and those with major congenital anomalies, chromosomal syndromes, or metabolic disorders that affect glucose homeostasis were excluded.

Ethical approval was obtained from the Institutional Review Board, and informed consent was waived owing to the retrospective nature of the study.

#### 2.1.1. Variables and Outcomes

Maternal variables, including age, hypertensive or diabetic disorders, chorioamnionitis, preterm premature rupture of membranes (PPROM), antenatal steroid exposure, body mass index, and glycated hemoglobin, and neonatal variables, such as birth weight (BW), head circumference, GA, sex, BW z-score, and delivery room interventions, were extracted from electronic records. The clinical parameters included the mode and duration of respiratory support, metabolic markers (FiO_2_, lactate, and base excess), insulin therapy, and postnatal steroid use. The major outcomes assessed were IVH, NEC, patent ductus arteriosus, ventilator-associated pneumonia (VAP), ROP, BPD, and neurodevelopmental outcomes at 18–24 months of corrected age.

#### 2.1.2. Definitions

BPD severity was classified according to Jensen’s 2019 criteria [18], based on respiratory support at 36 weeks postmenstrual age (PMA) (mild: nasal cannula ≤ 2 L/min; moderate: noninvasive support or cannula > 2 L/min; severe: invasive ventilation). Severe IVH was defined as Papile grade III–IV [19], and severe ROP was defined as stage ≥ 3 or requiring treatment [20]. Neurodevelopmental assessments were conducted at 18–24 months of corrected age using the Bayley Infant Neurodevelopmental Screener (BINS III) [21], a validated tool designed to identify infants at risk of developmental delays. BINS III evaluate cognitive, motor, and expressive/receptive language skills through structured tasks and caregiver-reported observations. Infants were assessed by trained personnel and categorized as follows, based on standardized cutoff scores: low-risk (scores within age expectations), moderate-risk (1–2 SD below the mean), or high-risk (>2 SD below the mean/failed critical items) for neurodevelopmental delay, enabling the identification of those who required early intervention.

#### 2.1.3. Measurement of Blood Glucose and Definition of Neonatal Hyperglycemia

Blood glucose monitoring followed a standardized unit protocol for EP infants and was performed at clinical discretion, typically 4–6 times daily during critical illness, initiation or advancement of parenteral nutrition, and insulin therapy, with additional measurements obtained for clinical indications such as glycosuria or instability. Blood glucose was measured in capillary or arterial whole blood using a point-of-care glucometer (Nova StatStrip Glucose/Hct Meter; Nova Biomedical, Waltham, MA, USA), maintained and calibrated according to manufacturer and institutional quality-control standards.

Neonatal hyperglycemia was defined as a peak blood glucose level > 8.3 mmol/L (>150 mg/dL) at any time during the first postnatal week. For each infant, the highest recorded value daily within the first 7 days determined exposure status and severity, categorized as mild (>8.3–≤10.0 mmol/L), moderate (>10.0–≤11.1 mmol/L), or severe (>11.1 mmol/L) [1].

#### 2.1.4. Statistical Analysis

Continuous variables are summarized as mean ± standard deviation (SD) and were compared using *t*-tests. Categorical variables are expressed as counts and percentages and were compared using the χ^2^ or Fisher’s exact test, as appropriate. Associations between neonatal hyperglycemia and outcomes were reported as odds ratios (ORs) or mean differences (MD) with 95% confidence intervals (CI). Statistical significance was defined as a two-sided *p*-value of <0.05. Analyses were performed using SPSS v30 (IBM Corp., Armonk, NY, USA).

To minimize confounding, propensity score matching was performed using a logistic regression model that included GA, BW, sex, antenatal steroid exposure, delivery room intubation, surfactant administration, and key perinatal factors known to influence both the glycemic status and neonatal outcomes. Infants with and without early hyperglycemia were matched 1:1 using nearest-neighbor matching without replacement, applying a caliper width of 0.1 of the logit of the propensity score. Incidence rate ratios (IRRs) and 95% confidence intervals were estimated using Poisson regression with a log offset for the number of ventilator days.

## 3. Results

### Cohort Characteristics

Among the 225 infants, 131 (58.2%) developed hyperglycemia during the first postnatal week, whereas 94 (41.8%) did not. Of the 131 infants, 21.4%, 42%, and 36.6% developed mild, moderate, and severe hyperglycemia, respectively. Insulin therapy was required in 13 patients with early neonatal hyperglycemia (9.9%). After propensity score matching, 109 infants remained in the matched cohort, including 55 with hyperglycemia and 54 without hyperglycemia.

Neonatal and characteristics are illustrated in Table 1. Before matching, infants with hyperglycemia were more premature (25.3 vs. 25.8 weeks, *p* = 0.004), had lower BW (803 g vs. 914 g, *p* < 0.001), and smaller head circumference (22.8 vs. 23.9 cm, *p* < 0.001). Maternal PPROM was significantly less frequent (20.6% vs. 33.0%, *p* = 0.036), and surfactant use in the delivery room was higher (63.4% vs. 50.0%, *p* = 0.045). After matching, none of these differences remained significant, indicating that the maternal and neonatal characteristics were balanced between the two groups. Other maternal and neonatal baseline demographic data did not differ significantly between the groups.

Table 2 summarizes the clinical differences between the two groups during the NICU course. Before matching, the infants in the hyperglycemia group had a more intensive respiratory course. Rescue treatment with high-frequency ventilation was significantly more common in the former group (44.5% vs. 20.5%; *p* < 0.001). The duration of invasive ventilation was longer (17.9 ± 23.2 vs. 6.1 ± 20.0 days; MD −11.8 days, 95% CI 5.3–183; *p* < 0.001). Early physiological markers were also worse, with a higher mean FiO_2_ during the first week (45.5 vs. 37.5; *p* = 0.007). Postnatal steroids (which were administered beyond the first postnatal week and were not used as a treatment for early hyperglycemia) were used more frequently in infants with hyperglycemia (20.2% vs. 5.1%; *p* = 0.003). Hyperglycemia persisting beyond the first week was also more frequent (19.5% vs. 3.4%, *p* < 0.001). Other variables, such as overall surfactant exposure during hospitalization, including surfactant administration after NICU admission, initial invasive ventilation rates, base excess, and discharge PMA, were not significantly different.

After matching, most early physiological differences disappeared. Rescue high-frequency ventilation, week-1 FiO_2_, and base excess were no longer significantly different between groups (all *p* > 0.20). However, the duration of invasive ventilation was longer in the hyperglycemia group (19.8 ± 25.3 vs. 8.9 ± 24.8 days; MD −10.9 days, *p* = 0.042). Postnatal steroid exposure remained higher (18.2% vs. 5.5%; *p* = 0.040), and persistent hyperglycemia beyond the first week was more common in the hyperglycemia group (27.3% vs. 6.1%; *p* = 0.004). Discharge PMA was similar across the groups.

The major morbidities and complications are presented in Table 3. Before matching, infants in the neonatal hyperglycemia group had markedly higher respiratory and ophthalmological morbidity. VAP was significantly more frequent in the hyperglycemia group (28.2% vs. 6.4%; OR 5.8, 95% CI 2.3–14.3; *p* < 0.001). Before matching, infants with hyperglycemia had a higher VAP incidence than those without (1.78 vs. 1.05 per 100 ventilator-days; Incidence Rate Ratio (IRR) 4.0, 95% CI 1.7–9.6; *p* = 0.001). This difference persisted after matching (1.45 vs. 0.37 per 100 ventilator-days; IRR 6.2, 95% CI 1.4–27.6; *p* = 0.016). Any ROP was also more common (66.9% vs. 46.5%; *p* = 0.003), as was severe ROP (26.3% vs. 10.5%; OR 3.0, 95% CI 1.4–6.8; *p* = 0.006). Similarly, the incidence of BPD was higher among infants with hyperglycemia (58.9% vs. 28.2%; OR 3.2, 95% CI 1.7–6.7; *p* < 0.001), as was moderate-to-severe BPD (46.8% vs. 22.2%; OR 3.0, 95% CI 2–5.8; *p* < 0.001). Other complications, including early- and late-onset sepsis, NEC, IVH (including severe IVH), pneumothorax, periventricular leukomalacia (PVL), patent ductus arteriosus (PDA), and PDA device closure, did not differ significantly between the groups.

After matching, most between-group differences were attenuated; however, two key associations remained. VAP remained significantly more frequent in the hyperglycemia group (23.6% vs. 3.7%; OR 8.0, 95% CI 1.7–377; *p* = 0.003), and severe ROP continued to occur more often (21.6% vs. 6.4%; OR 4.0, 95% CI 1.0–15.5; *p* = 0.032). After propensity score matching, no statistically significant differences were identified between the groups for any ROP, BPD, moderate-to-severe BPD, sepsis, NEC, IVH, pneumothorax, PVL, PDA, or PDA device closure. Although the point estimates for BPD and moderate-to-severe BPD were higher in the hyperglycemia group, these differences were not statistically significant.

Table 4 summarizes hospital stay, clinical outcomes, and additional morbidities. Before matching, infants with early neonatal hyperglycemia had a longer NICU admission (MD −17.8 days; 95% CI, 2.4–33.1; *p* = 0.024). Those infants were less likely to be discharged before 36 weeks PMA (13.9% vs. 36.7%; *p* < 0.001). The overall hospital stay was significantly longer (MD −24.9 days; *p* = 0.001). Death occurred at later postnatal ages in the hyperglycemia group (MD −25 days, 95% CI 1.4–48.5; *p* = 0.038), although the mortality rate did not differ significantly. Neurodevelopmental assessments were completed in 108/131 (82.4%) infants in the neonatal hyperglycemia group and 79/94 (84.0%) infants in the control group. Infants without follow-up data were excluded from the study. Hyperglycemia was associated with almost double the odds of any neurodevelopmental risk (49.1% vs. 32.9%; OR 1.96, 95% CI 1.1–3.6; *p* = 0.027). High-risk scores were more frequent, with odds 3.16 times higher (17.6% vs. 6.3%; OR 3.2, 95% CI 1.1–8.9; *p* = 0.023). Motor domain abnormalities (fine and gross motor) were also more frequent in the hyperglycemia group (25.9% vs. 13.9%; OR 2.2, 95% CI 1.0–4.7; *p* = 0.036). Other neurodevelopmental domains (cognitive, receptive, and expressive languages) showed no significant differences before matching the groups.

After matching, most of the differences were attenuated. NICU stay and discharge timing were no longer significantly different, although the age at death remained later in infants with neonatal hyperglycemia (MD −25.93 days, 95% CI 5.81–46.06; *p* = 0.015). Persistent differences in mortality rate, total hospital stay, and neurodevelopmental outcomes did not reach statistical significance after matching, including any neurodevelopmental risk, high-risk scores, or domain-specific delays. Overall, after balancing the baseline characteristics, only the age at death remained significantly different between the groups.

## 4. Discussion

In this cohort of EP infants, early neonatal hyperglycemia was observed in more than half of the population and was associated with adverse outcomes. Before adjustment, affected infants were smaller, less mature, and more clinically unstable, with greater respiratory support requirements, higher oxygen exposure, and biochemical evidence of metabolic stress, as reflected by elevated lactate levels. These associations remained after propensity score matching, which achieved a close balance across key baseline variables, including GA, BW, and early markers of clinical severity. Following adjustment, differences in the rates of VAP and severe ROP persisted and were not fully accounted for by the measured differences in prematurity or illness severity at baseline.

Our incidence of early neonatal hyperglycemia (58%) aligns with reports indicating that 20–80% of very preterm or extremely low birth weight infants develop neonatal hyperglycemia during the first week of life, especially within the context of aggressive parenteral nutrition and critical illness [3,22]. This common occurrence is further illustrated in case reviews and cohort studies [7]. Recent cohort studies and systematic reviews have shown that neonatal hyperglycemia is related to increased major morbidities and mortality, including IVH, ROP, NEC, and prolonged ventilation [23,24]. The association between early hyperglycemia, prolonged invasive ventilation, and increased postnatal steroid exposure reflects a more severe and sustained respiratory course in affected infants. Although early dysglycemia cannot be considered a causal determinant of BPD, it appears to cluster with clinical trajectories characterized by prolonged respiratory support and a greater inflammatory burden, which are well recognized contributors to BPD severity.

The strong and consistent correlation between early neonatal hyperglycemia and VAP in our cohort is a notable finding. After matching, infants with early neonatal hyperglycemia had approximately eight times higher odds of developing VAP. These findings are consistent with previous studies reporting an association between neonatal hyperglycemia and late-onset sepsis or culture-confirmed infections [25,26,27]. From a biological standpoint, hyperglycemia has been shown to impair innate immune function, including neutrophil chemotaxis, phagocytosis, cytokine signaling, and mucosal immunity [28,29]. In both adult and pediatric intensive care settings, hyperglycemia is widely recognized as a risk factor for nosocomial infections and VAP [30,31]. Our findings suggest a similar association in EP infants, who may be particularly susceptible because of prolonged mechanical ventilation and frequent, invasive monitoring.

Our study further supports the growing evidence that early neonatal hyperglycemia is linked to ROP, particularly in severe cases. Many observational cohorts and meta-analyses have shown an association between neonatal hyperglycemia and an increased risk of any ROP and ROP requiring treatment [9]. Kermorvant-Duchémin and colleagues showed that cumulative exposure above glycemic thresholds of 7–13 mmol/L (126–234 mg/dL) strongly predicts severe ROP [10]. Movsas et al. recently confirmed that early neonatal hyperglycemia increases the risk of severe ROP and suggested glucose-based thresholds for risk stratification [32]. Our matched analysis, demonstrating fourfold higher odds of severe ROP among hyperglycemic infants, aligns with these findings. The observed association between early neonatal hyperglycemia, higher early FiO_2_ requirements, and elevated lactate levels suggests that metabolic stress and impaired oxygen utilization frequently coexist in infants who later develop severe ROP. Rather than implying a direct pathogenic role of hyperglycemia, these findings support the concept that dysglycemia may act as a marker of systemic stress, which is known to disrupt retinal vascular development.

BPD is another significant outcome, and our findings align with the current evidence. Before matching, early neonatal hyperglycemia was associated with higher rates of BPD and moderate-to-severe BPD. After matching using Jensen’s 2019 support-based definition, the effect size diminished but remained clinically relevant, showing a trend toward an increased risk. The Jensen system classifies BPD severity based on respiratory support at 36 weeks PMA and strongly correlates with long-term respiratory and neurodevelopmental outcomes [18]. Our results indicate that although much of the connection between neonatal hyperglycemia and BPD is due to the overall illness severity, early dysglycemia may still biologically contribute through inflammatory and oxidative pathways. This perspective aligns with recent cohorts and meta-analyses [33,34] that emphasize the difficulty in completely distinguishing glycemic effects from underlying immaturity.

Unlike earlier studies that indicated higher mortality rates in hyperglycemic infants [6,22,35], our matched cohort showed that mortality rates did not differ significantly between groups after matching; however, infants with early hyperglycemia who died tended to do so at a later postnatal age. This pattern may reflect the impact of chronic morbidities, such as severe BPD or ROP, rather than early fatal instability. Improvements in neonatal supportive care may have reduced early mortality across groups, whereas longer-term complications continue to have an influence. Severe ROP and BPD are important factors connecting early physiological instability with later death and neurodevelopmental challenges [32,36], consistent with the profiles observed in our hyperglycemic infants.

The impact of early neonatal hyperglycemia on long-term neurodevelopment is uncertain. Prior to propensity score matching, infants with hyperglycemia exhibited higher overall neurodevelopmental risk, predominantly driven by motor domain impairment. These findings are consistent with previous reports associating early metabolic stress with compromised white matter development [9,10,37]. However, no statistically significant differences were observed between the groups in the cognitive domain. Although point estimates continued to favor the normoglycemic cohort, these differences did not reach statistical significance after adjustments. Such attenuation may reflect limitations related to the sample size, restricted follow-up duration, and reliance on early screening instruments. Evidence from continuous glucose monitoring studies further suggests that both hyperglycemia and glycemic variability contribute to neurological injury, including severe IVH and PVL [1,13,38,39]. Collectively, our findings support the concept that early neonatal hyperglycemia represents one component of a broader dysglycemic phenotype, indirectly associated with adverse neurodevelopmental trajectories through its relationship with severe chronic morbidity.

Finally, our findings highlight the importance of defining and managing neonatal hyperglycemia. In the first week, we used a threshold of >8.3 mmol/L (>150 mg/dL), and infants with neonatal hyperglycemia were more likely to experience persistent hyperglycemia beyond this period [1,25]. This was supported by Kermorvant-Duchémin and Movsas work [10,32], which showed that cumulative exposure to hyperglycemia above certain thresholds predicts severe ROP more accurately than isolated peaks. Other studies using continuous glucose monitoring suggest that glycemic variability may be equally important [1,38,40], suggesting that both burden and chronicity matter and that binary thresholds may underestimate the total risk. The feasibility of continuous glucose monitoring in VLBW infants is an area of growing interest [40].

From a therapeutic perspective, our findings support the need for careful prevention and monitoring rather than aggressive insulin-based tight glycemic control. Although early hyperglycemia identifies infants at higher risk for respiratory and ophthalmologic morbidity, randomized trials have not demonstrated improved survival or neurodevelopmental outcomes with early insulin therapy and have reported an increased hypoglycemia risk [2,35]. Current expert recommendations and clinical guidelines support optimizing glucose infusion rates, carefully titrating parenteral nutrition, and using continuous glucose monitoring, when available, to reduce periods of severe dysglycemia [1,14,16,17,41]. Our findings support this balanced approach, as early neonatal hyperglycemia identifies a high-risk phenotype with greater respiratory and retinal morbidity but not an independent mortality signal that would justify aggressive insulin therapy [2].

## 5. Strengths and Limitations

The major strengths of this study include a large, contemporary cohort of extremely preterm infants, standardized ROP screening and BPD classification, and the use of propensity score matching. Limitations include the single-center, retrospective design, potential residual confounding, reliance on intermittent glucose measurements, use of early neurodevelopmental screening, missing data for several clinically important variables, and the absence of detailed timing data for postnatal steroid exposure. These factors warrant cautious interpretation of the results, as some covariates remained unbalanced after matching and propensity score matching cannot account for unmeasured confounding, such as early illness severity.

## 6. Conclusions

Early neonatal hyperglycemia in a matched cohort of EP infants was associated with increased ventilator-associated pneumonia, longer duration of invasive ventilation, higher postnatal steroid exposure, and severe ROP. These relationships likely reflect a high-risk clinical phenotype characterized by greater early respiratory and metabolic stress levels. Although causality cannot be inferred from this retrospective observational study, early dysglycemia may help identify infants who require closer monitoring and targeted preventive strategies. Future prospective studies should evaluate whether specific glucose management approaches improve clinically meaningful outcomes without increasing the risk of hypoglycemia.

## Figures and Tables

**Table 1:** Maternal and Neonatal Characteristics.

Variable	Before Matching				After Matching			
	No Neonatal Hyperglycemia (*n* = 94)	Neonatal Hyperglycemia (*n* = 131)	OR/MD (95% CI)	*p*-Value	No Neonatal Hyperglycemia (*n* = 54)	Neonatal Hyperglycemia (*n* = 55)	OR/MD (95% CI)	*p*-Value
Maternal								
Hypertension, *n* (%)	12 (13.8%)	15 (11.8%)	0.8 (0.4–1.9)	0.668	11 (20.4%)	5 (9.1%)	0.4 (0.1–1.2)	0.096
Hyperglycemia/Diabetes, *n* (%)	15 (16.0%)	19 (14.8%)	0.9 (0.4–1.9)	0.820	7 (13%)	6 (11.1%)	0.8 (0.3–2.7)	0.767
Chorioamnionitis, *n* (%)	17 (18.1%)	16 (12.2%)	0.6 (0.3–1.3)	0.220	6 (11.1%)	10 (18.2%)	1.8 (0.6–5.3)	0.297
PPROM, *n* (%)	31 (33.0%)	27 (20.6%)	0.5 (0.3–0.9)	0.036	12 (22.2%)	14 (25.5%)	1.2 (0.5–2.9)	0.692
Antenatal steroids, *n* (%)	76 (80.9%)	114 (87.0%)	1.6 (0.8–3.3)	0.208	42 (77.8%)	48 (87.3%)	2 (0.7–5.4)	0.191
Maternal BMI (kg/m^2^)	29.9 ± 6.6	28.9 ± 5.4	0.9 (−0.7–2.5)	0.253	31.5 ± 7.7	29.2 ± 6.0	2.3 (−0.4–4.9)	0.098
Maternal HbA1c (%)	5.3 ± 0.5	5.3 ± 0.4	−0.04 (−0.2–0.1)	0.658	5.1 ± 0.2	5.3 ± 0.4	−0.2 (−0.4–0.01)	0.070
Neonatal								
SGA, *n* (%)	3 (3.2%)	8 (6.1%)	2 (0.5–7.6)	0.367	3 (5.6%)	1 (1.8%)	0.3 (0.03–3.1)	0.363
LGA, *n* (%)	11 (11.7%)	13 (9.9%)	0.8 (0.4–1.9)	0.670	4 (7.4%)	3 (5.5%)	0.7 (0.2–3.4)	0.716
Male gender, *n* (%)	53 (56.4%)	73 (55.7%)	1 (0.6–1.7)	0.922	29 (53.7%)	26 (47.3%)	0.8 (0.4–1.6)	0.502
Birth weight (g)	913.8 ± 187.2	803.0 ± 164.3	110.8 (64.4–157.2)	<0.001	885.5 ± 169.6	850.2 ± 159.3	35.3 (−27.2–97.8)	0.266
Gestational age (weeks)	25.8 ± 1.16	25.3 ± 1.3	0.5 (0.2–0.8)	0.004	25.9 ± 1.1	25.6 ± 1.3	0.3 (−0.2–0.7)	0.218
Head circumference (cm)	24 ± 1.8	22.8 ± 1.8	1.1 (0.6–1.7)	<0.001	23.8 ± 1.9	23.4 ± 2.1	0.4 (−0.4–1.2)	0.312
BW z-score	0.3 ± 0.9	0.01 ± 0.9	0.3 (0.1–0.5)	0.013	0.2 ± 0.9	0.1 ± 0.7	0.04 (−2.7–0.4)	0.777
Intubation in DR, *n* (%)	69 (73.4%)	106 (80.9%)	1.5 (0.8–2.9)	0.181	45 (83.3%)	40 (72.7%)	0.5 (0.2–1.4)	0.182
Surfactant in DR, *n* (%)	47 (50.0%)	83 (63.4%)	1.7 (1.0–3)	0.045	26 (48.1%)	29 (52.7%)	1.2 (0.6–2.5)	0.633

This table compares the baseline characteristics of mothers and their extremely preterm infants with and without neonatal hyperglycemia, both before and after propensity score matching. Continuous variables are presented as mean ± standard deviation, and categorical variables are presented as numbers (percentages). Effect estimates were reported as odds ratios (ORs) for categorical variables and mean differences (MD) for continuous variables, each with 95% confidence intervals (CIs). *p*-values reflect between-group comparisons before and after matching. CI, confidence interval; PPROM, preterm premature rupture of membranes; BMI, body mass index; HbA1c, glycated hemoglobin; SGA, small for gestational age; LGA, large for gestational age; BW, birth weight; DR, delivery room. Missing data: maternal hypertension (*n* = 11), maternal BMI (*n* = 7), maternal Hb A1C (*n* = 113), and head circumference (*n* = 42).

**Table 2:** Comparison of Clinical Parameters in the NICU.

Variables	Before Matching				After Matching			
	No Neonatal Hyperglycemia (*n* = 94)	Neonatal Hyperglycemia (*n* = 131)	OR/MD (95% CI)	*p*-Value	No Neonatal Hyperglycemia (*n* = 54)	Neonatal Hyperglycemia (*n* = 55)	OR/MD (95% CI)	*p*-Value
Any surfactant, *n* (%)	73 (82.0%)	114 (89.1%)	1.8 (0.8–3.9)	0.139	52 (96.3%)	48 (87.3%)	0.26 (0.1–1.3)	0.161
Invasive ventilation, *n* (%)	76 (85.4%)	119 (93.0%)	2.3 (0.92–5.54)	0.069	52 (96.3%)	51 (92.7%)	0.5 (0.1–2.8)	0.679
High frequency ventilation (rescue), *n* (%)	18 (20.5%)	57 (44.5%)	3.1 (1.7–5.8)	<0.001	10 (18.5%)	15 (27.3%)	1.6 (0.7–4.1)	0.277
Invasive ventilation, days	6.1 ± 20.0	17.9 ± 23.2	−11.8 (−18.3 to −5.3)	<0.001	8.9 ± 24.8	19.8 ± 25.3	−10.9 (−21.5 to −0.4)	0.042
Highest FiO_2_ (first week), %	37.5 ± 21.6	45.5 ± 22.0	−8.1 (−13.9 to −2.0)	0.007	37.9 ± 20.2	35.9 ± 18.5	1.9 (−5.5–9.3)	0.602
Highest lactate (first week), mmol/L	3.6 ± 3.7	4.9 ± 5.0	−1.3 (−2.5 to −0.02)	0.045	3.4 ± 3.2	3.1 ± 2.1	0.3 (−0.8–1.3)	0.623
Highest Base Excess (first week), mmol/L	7.9 ± 4.5	10.4 ± 12.0	−2.5 (−5.2–0.1)	0.063	7.9 ± 4.7	7.8 ± 3.1	0.1 (−1.4–1.6)	0.882
Postnatal steroids, *n* (%)	4 (5.1%)	22 (20.2%)	4.7 (1.6–14.4)	0.003	3 (5.5%)	10 (18.2%)	4.6 (1.6–14.4)	0.040
Discharge PMA, weeks	39.9 ± 7.3	41.8 ± 6.9	−2 (−4.2 to −0.2)	0.073	38.5 ± 5.1	40.9 ± 7.8	−2.4 (−5.3–0.4)	0.090
Hyperglycemia beyond the first week of life, *n* (%)	3 (3.4%)	25 (19.5%)	6.8 (2–23.3)	<0.001	3 (6.1%)	15 (27.3%)	5.8 (1.6–21.3)	0.004

FiO_2_ fraction of inspired oxygen, PMA postmenstrual age. Missing data: surfactant administration (*n* = 8), ventilation (*n* = 8), high-frequency ventilation (*n* = 8), ventilation days (*n* = 36), postnatal steroids (*n* = 37), discharge PMA (*n* = 51), and hyperglycemia beyond 1 week (*n* = 10).

**Table 3:** Major Morbidities and Complications.

Variables	Before Matching				After Matching			
	No Neonatal Hyperglycemia (*n* = 94)	Neonatal Hyperglycemia (*n* = 131)	OR/MD (95% CI)	*p*-Value	No Neonatal Hyperglycemia (*n* = 54)	Neonatal Hyperglycemia (*n* = 55)	OR/MD (95% CI)	*p*-Value
Early onset sepsis, *n* (%)	2 (2.1%)	1 (0.8%)	0.4 (0.03–4)	0.573	1 (1.9%)	0 (0.0%)	0.5 (0.4–0.6)	0.495
Late onset sepsis, *n* (%)	13 (13.8%)	23 (17.6%)	1.3 (0.6–2.8)	0.452	10 (18.5%)	16 (29.1%)	1.8 (0.7–4.4)	0.195
First sepsis episode DOL, days	16.3 ± 12.1	20.6 ± 17	−4.3 (−14.5–5.9)	0.4	16.6 ± 12.6	20.3 ± 14.6	−3.6 (−14.7–7.6)	0.512
UTI, *n* (%)	2 (2.1%)	5 (3.8%)	1.8 (0.3–9.6)	0.702	0 (0%)	1 (1.8%)	0.5 (0.4–0.60)	0.100
IVH, *n* (%)	19 (20.2%)	39 (30%)	1.7 (0.9–3.2)	0.099	12 (22.2%)	16 (29.1%)	1.4 (0.6–3.4)	0.412
Severe IVH, *n* (%)	2 (2.1%)	9 (6.9%)	3.4 (0.7–16.2)	0.125	2 (3.7%)	3 (5.5%)	1.5 (0.24–9.4)	0.100
NEC, *n* (%)	4 (4.5%)	11 (8.6%)	2 (0.6–6.4)	0.250	3 (5.6%)	6 (10.9%)	2.1 (0.5–8.8)	0.489
VAP, *n* (%)	6 (6.4%)	37 (28.2%)	5.8 (2.3–14.3)	<0.001	2 (3.7%)	13 (23.6%)	8.0 (1.7–37.7)	0.003
VAP/100 ventilation days, *n*	1.05	1.78	4.0 (1.7–9.6) *	0.001	0.37	1.45	6.2 (1.4–27.6) *	0.016
Pneumothorax, *n* (%)	2 (2.2%)	8 (6.3%)	2.90 (0.60–13.99)	0.204	1 (1.9%)	1 (1.8%)	1 (0.1–16.1)	0.100
PVL, *n* (%)	1 (1.1%)	6 (4.7%)	4.5 (0.5–37.7)	0.243	1 (1.9%)	2 (3.7%)	2.0 (0.2–23.2)	0.100
PDA, *n* (%)	44 (52.4%)	76 (62.8%)	1.5 (0.9–2.7)	0.136	25 (52.1%)	25 (50.0%)	0.9 (0.4–2.0)	0.837
ROP, *n* (%)	40 (46.5%)	79 (66.9%)	2.3 (1.3–4.1)	0.003	21 (44.7%)	28 (54.9%)	1.5 (0.7–3.3)	0.312
Severe ROP, *n* (%)	9 (10.5%)	31 (26.3%)	3.0 (1.4–6.8)	0.005	3 (6.4%)	11 (21.6%)	4.0 (1.0–15.5)	0.032
BPD, *n* (%)	23 (28.2%)	66 (58.9%)	3.2 (2–6.7)	<0.001	11 (25.0%)	21 (43.8%)	2.3 (1–5.7)	0.059
Moderate–severe BPD, *n* (%)	18 (22.2%)	52 (46.4%)	3.0 (1.6–5.8)	<0.001	7 (15.9%)	16 (33.3%)	2.6 (1–7.2)	0.054
PDA device closure, *n* (%)	5 (6.9%)	6 (5.8%)	0.8 (0.2–2.8)	0.762	2 (4.8%)	2 (4.3%)	0.9 (0.1–6.8)	0.100

DOL, day of life; UTI, urinary tract infection; IVH, intraventricular hemorrhage; NEC, necrotizing enterocolitis; VAP, ventilator-associated pneumonia; PVL, periventricular leukomalacia; PDA, patent ductus arteriosus; ROP, retinopathy of prematurity; BPD, bronchopulmonary dysplasia. * Incidence rate ratios (IRRs) with 95% confidence intervals were estimated using Poisson regression with a log offset for ventilator-days. Missing data: first sepsis DOL (*n* = 107), PDA (*n* = 20), PDA device closure (*n* = 50), and BPD (*n* = 32).

**Table 4:** Hospital Stay, Clinical Outcomes, and Morbidity.

Variables	Before Matching				After Matching			
	No Neonatal Hyperglycemia (*n* = 94)	Neonatal Hyperglycemia (*n* = 131)	OR/MD (95% CI)	*p*-Value	No Neonatal Hyperglycemia (*n* = 54)	Neonatal Hyperglycemia (*n* = 55)	OR/MD (95% CI)	*p*-Value
NICU stay, days	97.7 ± 51.4	115.5 ± 49.8	−17.8 (−33.1 to −2.4)	0.024	88.2 ± 36.1	107.3 ± 53.1	−19.0 (−38.4–0.4)	0.055
Discharge before 36 weeks PMA, *n* (%)	29 (36.7%)	15 (13.9%)	0.3 (0.1–0.6)	<0.001	14 (32.6%)	9 (18.8%)	0.5 (0.2–1.3)	0.130
Discharge after 36 weeks, *n* (%)	52 (64.2%)	94 (85.5%)	3.3 (1.6–6.6)	<0.001	30 (68.2%)	38 (79.2%)	1.8 (0.7–4.6)	0.231
Discharge PMA, weeks	39.9 ± 7.3	41.8 ± 7	−2 (−4.2 to −0.2)	0.073	38.5 ± 5.13	40.9 ± 7.8	−2.4 (−5.3–0.4)	0.090
Death, *n* (%)	13 (13.8%)	21 (16.0%)	1.2 (0.6–2.5)	0.649	10 (18.5%)	7 (12.7%)	0.6 (0.2–1.8)	0.405
Death DOL, days	12.1 ± 12.1	37.1 ± 40.3	−25 (−48.5 to −1.4)	0.038	13.5 ± 13.5	39.4 ± 25.4	−25.9 (−46.1 to −5.8)	0.015
Hospital stays, days	90.5 ± 30.6	115.3 ± 60	−24.9 (−39.5 to −10.2)	<0.001	88.2 ± 36.1	107.3 ± 53.1	−19.0 (−38.5–0.4)	0.055
Any neurodevelopmental risk, *n* (%)	26 (32.9%)	53 (49.1%)	2 (1.1–3.6)	0.027	15 (34.9%)	21 (43.8%)	1.5 (0.6–3.4)	0.388
High neurodevelopmental, *n* (%)	5 (6.3%)	19 (17.6%)	3.2 (1.1–8.9)	0.023	2 (4.7%)	5 (10.4%)	2.4 (0.4–13)	0.265
Cognitive, *n* (%)	6 (7.6%)	9 (8.3%)	1.1 (0.4–3.2)	0.854	3 (7.0%)	1 (2.1%)	0.3 (0.02–2.8)	0.268
Neurologic (fine and gross motor), *n* (%)	11 (13.9%)	28 (25.9%)	2.2 (1.0–4.7)	0.046	6 (14.0%)	11 (22.9%)	1.8 (0.6–5.5)	0.273
Receptive language, *n* (%)	15 (19.0%)	16 (14.8%)	0.7 (0.3–1.6)	0.449	9 (20.9%)	8 (16.7%)	0.8 (0.3–2.2)	0.602
Expressive language, *n* (%)	20 (25.3%)	37 (34.3%)	1.5 (0.8–2.9)	0.189	11 (23.4%)	15 (30.0%)	1.4 (0.6–3.5)	0.464

Missing data: stay days (*n* = 51), hospital stays (*n* = 38), discharge before 36 weeks (*n* = 38), discharge after 36 weeks (*n* = 34), neurodevelopmental assessment (death, *n* = 33; missing assessment, *n* = 5).

## Data Availability

The data underlying this study are stored at Hamad Medical Corporation and contain confidential patient information. Owing to institutional and ethical restrictions, the dataset cannot be made publicly available. De-identified data may be obtained from the corresponding author upon reasonable request following approval from the Institutional Review Board.

## Abbreviations

BINS	Bayley Infant Neurodevelopmental Screener
BPD	Bronchopulmonary Dysplasia
CI	Confidence Interval
DOL	Day of Life
DR	Delivery Room
EP	Extremely Preterm
IVH	Intraventricular Hemorrhage
NEC	Necrotizing Enterocolitis
NICU	Neonatal Intensive Care Unit
OR	Odds Ratio
PDA	Patent Ductus Arteriosus
PMA	Postmenstrual Age
PVL	Periventricular Leukomalacia
ROP	Retinopathy of Prematurity
SD	Standard Deviation
SMD	Standardized Mean Difference
VAP	Ventilator-Associated Pneumonia
